# Progress in Immunization Safety Monitoring — Worldwide, 2020–2022

**DOI:** 10.15585/mmwr.mm7249a2

**Published:** 2023-12-08

**Authors:** Erin F. Blau, Madhava Ram Balakrishnan, Helena Sköld, Ravi Shankar Santhana Gopala Krishnan, Pinelopi Lundquist, Shanthi Pal, Jane F. Gidudu

**Affiliations:** ^1^Global Immunization Division, Center for Global Health, CDC; ^2^Department of Regulation and Prequalification, World Health Organization, Geneva, Switzerland; ^3^Uppsala Monitoring Centre, WHO Collaborating Centre for International Drug Monitoring, Uppsala, Sweden; ^4^Department of Data and Analytics, World Health Organization, Geneva, Switzerland.

**Summary**

**What is already known about this topic?**

In 2020, the World Health Organization (WHO) recommended a new case-based vaccine safety monitoring indicator: one or more serious adverse events following immunization (AEFIs) per 1 million total population per year.

**What is added by this report?**

In 2022, 92 (43%) of 215 WHO-affiliated countries and territories achieved the new case-based indicator. During 2020–2022, four of six WHO regions reported an increase in joint reporting of national AEFI data from national regulatory authorities (NRAs) and national expanded programs on immunization (EPIs).

**What are the implications for public health practice?**

Case-based reporting promotes timely AEFI detection, reporting, investigation, and response by NRAs and EPIs. Improving case-based data sharing globally can provide valuable insights into trends and regional characteristics of serious AEFI.

## Abstract

Effective surveillance of adverse events following immunization (AEFIs) primarily relies on the collaboration of two partners: national regulatory authorities (NRAs) and national expanded programs on immunization (EPIs). In December 2020, the World Health Organization (WHO) Global Advisory Committee for Vaccine Safety recommended a new case-based indicator of national capacity to monitor immunization safety: at least one serious AEFI reported per 1 million total population per year. To achieve this indicator, WHO-affiliated countries and territories (WHO countries) rely upon data generated from functional AEFI surveillance systems. This report describes 2020–2022 global, regional, and national progress in use of the newly introduced immunization safety monitoring indicator and progress on joint AEFI reporting from national EPIs and NRAs. Among WHO countries, 51 (24%) of 214 implemented the new indicator in 2020, 111 (52%) of 214 implemented it in 2021, and 92 (43%) of 215 in 2022. In 2020, 41 (19%) WHO countries reported AEFI data jointly from EPIs and NRAs; this increased to 55 (26%) in 2021 and 57 (27%) in 2022. These findings, resulting in part from the intensified support for COVID-19 vaccination, demonstrate that national AEFI surveillance systems increasingly support the timely use and sharing of case-based immunization safety data, but work is still needed to strengthen global vaccine safety monitoring.

## Introduction

Robust postauthorization and postlicensure immunization safety monitoring systems help ensure that the benefits of vaccination continue to outweigh the risks. During the previous decade, global progress was made in achieving at least minimum functionality of immunization safety monitoring through the establishment of national immunization safety surveillance systems. In December 2014, the World Health Organization (WHO) established the first indicator of minimal national vaccine safety surveillance as aggregate reporting of more than 10 adverse events following immunization (AEFIs) per 100,000 surviving infants (*1*). In 2019, this indicator was achieved by 121 (57%) of 214 WHO-affiliated countries and territories (WHO countries).* The WHO Vaccine Safety Blueprint 2.0 highlighted the need for more comprehensive indicators for national, regional, and global safety surveillance systems (*2*,*3*). Subsequently, in December 2020, WHO’s Global Advisory Committee for Vaccine Safety recommended the adoption of a new case-based indicator for monitoring progress in AEFI surveillance for all age groups: the number of serious^†^ AEFIs reported per 1 million total national or subnational population in a year (*4*,*5*). This case-based reporting indicator was proposed to facilitate accurate AEFI reporting and increase national system sensitivity in detecting vaccine safety signals.^§^

In many WHO countries, effective AEFI surveillance relies on the collaboration of two national partners: 1) national regulatory authorities (NRAs), which are national organizations responsible for ensuring that pharmaceuticals and biologics are properly evaluated and that they meet international standards of quality, safety, and efficiency,^¶^ and 2) national expanded programs on immunization (EPIs). EPIs typically oversee national procurement, storage, and delivery of vaccines, including the staffing and training of health care workers responsible for administering vaccines and caring for patients reporting AEFIs. As a result, EPIs play an important role in identifying and reporting AEFIs. NRAs are mandated to perform postauthorization and postlicensure AEFI surveillance and must work in tandem with EPIs to support health care–worker training and management of AEFI reports and investigations, including support for independent assessments of causality for serious AEFIs. Coordination of AEFI reporting among EPIs and NRAs improves data quality, completeness, and usability, so that safety signals can be detected and identified quickly (*6*,*7*). This article updates a previous report (*6*), introduces WHO’s new indicator for vaccine safety monitoring, and describes progress with national-level coordination and cooperation among two national partners in AEFI reporting.

## Methods

WHO countries meeting the newly recommended global immunization safety monitoring indicator were identified using national AEFI data reported to VigiBase, WHO’s global pharmacovigilance database for individual case reports of suspected adverse reactions to medicinal products, including vaccines (*8*). AEFI reports in VigiBase were classified as serious based on information with AEFI reporting forms and associated case investigation forms used by WHO countries.

Coordination of AEFI reporting among national EPI and NRA programs was measured annually based on response to the following question in the WHO and UNICEF electronic Joint Reporting Form, a questionnaire for the passive joint collection of aggregate AEFI data: “What is the source of data for the total number of serious adverse events reported?” Possible responses included “EPI only,” “NRA only,” “both EPI and NRA,” or “other” (*9*,*10*).

National reporting to VigiBase and the Joint Reporting Form is voluntary and varies by year. WHO countries not reporting to these systems during the reporting period (2020–2022) were considered as not meeting the requirements for either the newly recommended indicator or coordination of EPI and NRA AEFI reporting; however, these countries were included in the denominator when calculating percentages. Geographic areas are reported by WHO country (214 in 2020 and 2021; 215 in 2022)** and WHO region: African Region (AFR),^††^ Region of the Americas (AMR),^§§^ Eastern Mediterranean Region (EMR),^¶¶^ European Region (EUR),*** South-East Asia Region (SEAR),^†††^ and Western Pacific Region (WPR).^§§§^ This activity was reviewed by CDC, deemed not research, and was conducted consistent with applicable federal law and CDC policy.^¶¶¶^

## Results

### Indicator Data Reporting

**FIGURE 1:**
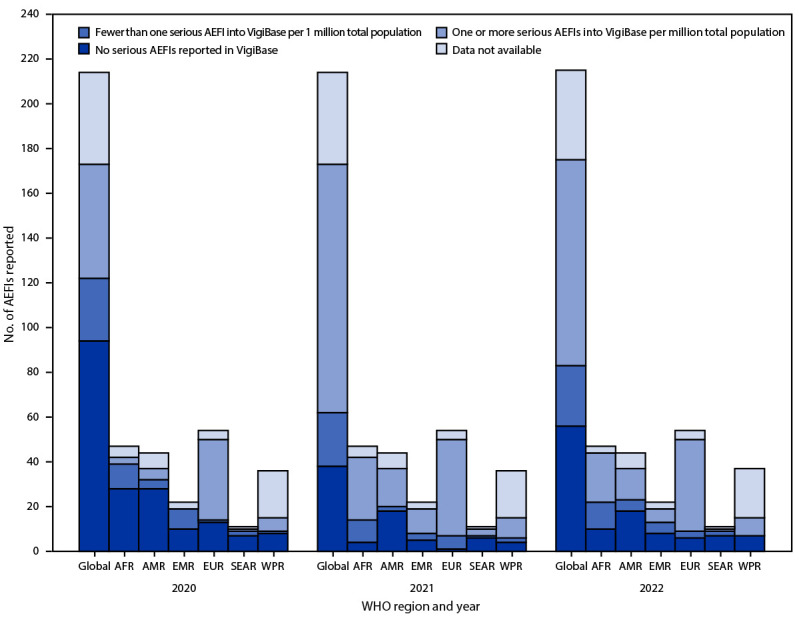
World Health Organization–affiliated countries and territories reporting serious adverse events following immunization into VigiBase,* by World Health Organization region — worldwide, 2020–2022 **Abbreviations**: AEFIs = adverse events following immunization; AFR = African Region; AMR = Region of the Americas; EMR = Eastern Mediterranean Region; EUR = European Region; SEAR = South-East Asia Region; WHO = World Health Organization; WPR = Western Pacific Region. * VigiBase is WHO's global pharmacovigilance database for individual case reports of suspected adverse reactions to medicinal products, including vaccines. https://www.who-umc.org/vigibase/

During 2020, 2021, and 2022, a total of 51 (24%) of 214, 111 (52%) of 214, and 92 (43%) of 215 WHO countries, respectively, achieved the new safety monitoring indicator (i.e., number of serious AEFIs reported per 1 million total national or subnational population in a year). During these same years, 79 (37%), 135 (63%), and 118 (55%) WHO countries, respectively, reported any serious AEFI data to VigiBase (Figure 1) (Supplementary Figure 1, https://stacks.cdc.gov/view/cdc/135986). In 2022, the region with the highest proportion of WHO countries meeting the new indicator was EUR (76%), followed by AFR (47%), AMR (32%), EMR (27%), and WPR (22%); the region with the lowest proportion was SEAR (9%). The largest increase in the number and percentage of WHO countries meeting the new indicator occurred in AFR, where the number of WHO countries meeting the indicator increased more than eightfold, from three (6%) in 2020, to 28 (60%) in 2021, but subsequently declined 21%, to 22 (47%) in 2022. Whereas all WHO regions except SEAR observed an overall increase in the number and percentage of WHO countries achieving the new indicator from 2020 to 2022, a decrease was observed in every region from 2021 to 2022 (Table).

**TABLE:** World Health Organization–affiliated countries and territories* reporting at least one serious adverse event following immunization per 1 million total population into VigiBase,^†^ by World Health Organization region — worldwide, 2020–2022

WHO region	No. of WHO-affiliated countries	Yr, no. (%)		
		2020	2021	2022
AFR	47	3 (6.4)	28 (59.6)	22 (46.8)
AMR	44	5 (11.4)	17 (38.6)	14 (31.8)
EMR	22	0 (—)	11 (50.0)	6 (27.3)
EUR	54	36 (66.7)	43 (79.6)	41 (75.9)
SEAR	11	1 (9.1)	3 (27.3)	1 (9.1)
WPR	36 (2020–2021)	6 (16.7)	9 (25.0)	8 (22.2)
	37 (2022)^§^			
**All regions**	**214 (2020–2021)**	**51 (23.8)**	**111 (51.9)**	**92 (42.8)**
	**215 (2022)**			

**Abbreviations:** AFR = African Region; AMR = Region of the Americas; EMR = Eastern Mediterranean Region; EUR = European Region; SEAR = South-East Asia Region; WHO = World Health Organization; WPR = Western Pacific Region.

* Members of WHO are grouped according to regional distribution (194 countries and 21 territories). All countries that are members of the United Nations can become members of WHO by accepting its constitution. Other countries can be admitted as members when their application has been approved by a simple majority vote of the World Health Assembly. Territories that are not responsible for the conduct of their international relations can be admitted as associate members upon application made on their behalf by the WHO member country or other authority responsible for their international relations.

† VigiBase is WHO's global pharmacovigilance database for individual case reports of suspected adverse reactions to medicinal products, including vaccines. https://www.who-umc.org/vigibase/

^§^ In 2022, Pitcairn Islands was added as a WHO territory in WPR, increasing the total number of WHO-affiliated countries from 214 to 215, and in WPR from 36 to 37.

### Sources of Indicator Data

**FIGURE 2:**
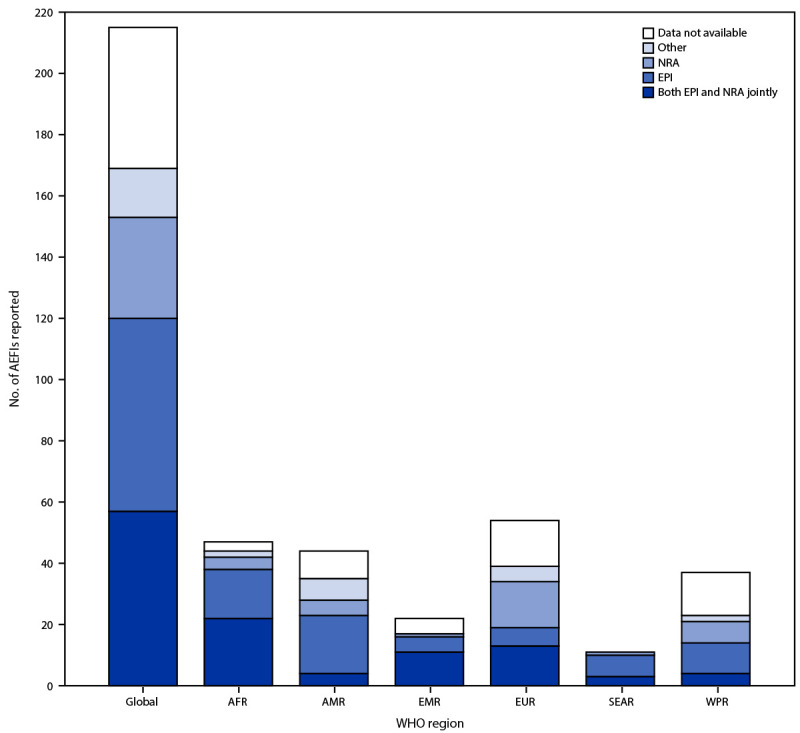
Number of adverse events following immunization reported on the World Health Organization/UNICEF Joint Reporting Form, by data source and World Health Organization region — worldwide, 2022 **Abbreviations**: AEFIs = adverse events following immunization; AFR = African Region; AMR = Region of the Americas; EMR = Eastern Mediterranean Region; EPI = expanded program on immunization; EUR = European Region; NRA = national regulatory authority; SEAR = South-East Asia Region; WHO = World Health Organization; WPR = Western Pacific Region.

In 2022, 169 (79%) of 215 WHO countries reported the source of national AEFI data; the primary data source was EPI for 63 (29%) countries, NRA for 33 (15%), and both EPI and NRA for 57 (27%) (Figure 2) (Supplementary Figure 2, https://stacks.cdc.gov/view/cdc/135987). Seventeen (8%) WHO countries **** reported other independent sources for national AEFI data (e.g., the Vaccine Adverse Event Reporting System in the United States) in 2022. During the reporting period, among six WHO regions, the percentage of countries reporting both EPI and NRA as the primary source of national AEFI data increased in four (AFR, EMR, EUR, and SEAR), decreased in AMR, and remained unchanged in WPR.

## Discussion

Compared with 2020, most WHO regions made progress toward achieving the two immunization safety monitoring measures in 2021 and 2022, by attaining the Global Advisory Committee for Vaccine Safety’s indicator of reporting at least one serious AEFI per 1 million total population per year, and by jointly reporting AEFI data from EPIs and NRAs. Progress has been particularly notable in AFR and EMR, where WHO has continued to support vaccine safety training and the development of standardized data collection tools and national AEFI surveillance system guidelines. Despite this progress, however, all WHO regions continue to report low percentages of countries jointly reporting EPI and NRA AEFI data; although EMR achieved the highest regional percentage of WHO countries jointly reporting EPI and NRA AEFI data, only 50% of countries in the EMR reported both. Fewer than one half, 92 (43%) of 215 WHO countries are currently meeting the target for the new safety monitoring indicator, and only in EUR are more than one half of countries reporting, demonstrating that additional work is needed to strengthen global vaccine safety monitoring.

The recent COVID-19 pandemic response and subsequent national immunization activities likely contributed substantially to the progress in global immunization safety monitoring, in large part because of increased funding and provision of intensified technical support from global partners. With nationally focused activities to increase COVID-19 vaccine distribution and vaccination coverage paired with innovative vaccine safety monitoring approaches (e.g., smartphone applications), the highest proportion of WHO countries meeting the new indicator was observed in 2021. Most AEFI cases reported in 2021 were associated with COVID-19 vaccines, reinforcing that case-based data from national AEFI surveillance systems can be shared globally (i.e., to VigiBase). Despite these gains, a slight decrease was observed in the proportion of WHO countries meeting the new reporting indicator in many WHO regions during 2022, likely because of a decline in national COVID-19 vaccination campaigns and less intensive AEFI surveillance. The current findings indicate that further measures are needed to strengthen global vaccine safety monitoring though technical support, standardized tools, and guidelines, and that better approaches to promote nationally coordinated AEFI reporting among EPIs and NRAs are needed.

### Limitations

The findings in this report are subject to at least three limitations. First, this report relied only on data submitted to VigiBase to determine progress toward meeting the new AEFI surveillance indicator. Reporting to VigiBase is voluntary and varies by year. Some WHO countries might not consistently submit data to VigiBase and thus are not identified as meeting the AEFI surveillance indicator during the reporting period. Second, because of the distinct roles of reporting to VigiBase by NRAs and to the Joint Reporting Form by EPIs, assessment of the role the relationship among NRAs and EPIs plays in meeting the new immunization safety monitoring indicator was not possible. Finally, whereas other factors contribute to national capacity to develop and maintain an immunization safety system, this report focused on only two immunization safety measures: the new case-based indicator and the reporting source of AEFI data, which might not reflect the actual functionality of a national immunization safety surveillance system.

### Implications for Public Health Practice

A shift to case-based reporting enables and promotes the use of AEFI data for action, including timely detection, reporting, investigation, and causality assessment by national AEFI committees, and response to reported serious AEFIs or clusters by national EPIs and NRAs. In addition, when shared globally, individual case safety reports can collectively contribute to the description of trends and regional characteristics of rare, but serious, AEFIs that might be difficult to detect through national aggregate data. Continued efforts in capacity building of immunization safety monitoring systems are needed to ensure and promote public confidence in national vaccination programs.

## Footnotes

* WHO-affiliated countries (194) and states and territories (21).

† Seriousness is based on patient or event outcome or action criteria and defines regulatory reporting obligations. An AEFI is considered serious if it results in death, is life-threatening, requires inpatient hospitalization or prolongation of existing hospitalization, results in persistent or substantial disability or incapacity, or is a congenital anomaly or birth defect. Other situations could also be considered serious after the application of medical and scientific judgment. The application of the criteria is dependent on its interpretation and health practices in a particular setting.

§ A vaccine safety signal is information that indicates a potential link between a vaccine and an event previously unknown or incompletely documented that might affect health.

¶
https://www.who.int/southeastasia/activities/national-regulatory-agencies#:~:text

** In 2022, Pitcairn Islands was added as a WHO territory in WPR, increasing the total number of WHO countries from 214 to 215, and in WPR from 36 to 37.

†† WHO countries in AFR (47): Algeria, Angola, Benin, Botswana, Burkina Faso, Burundi, Cabo Verde, Cameroon, Central African Republic, Chad, Comoros, Côte d'Ivoire, Democratic Republic of the Congo, Equatorial Guinea, Eritrea, Eswatini, Ethiopia, Gabon, The Gambia, Ghana, Guinea, Guinea-Bissau, Kenya, Lesotho, Liberia, Madagascar, Malawi, Mali, Mauritania, Mauritius, Mozambique, Namibia, Niger, Nigeria, Republic of the Congo, Rwanda, Sao Tome and Principe, Senegal, Seychelles, Sierra Leone, South Africa, South Sudan, Togo, Uganda, Tanzania, Zambia, and Zimbabwe.

§§ WHO countries in AMR (35): Antigua and Barbuda, Argentina, Bahamas, Barbados, Belize, Bolivia, Brazil, Canada, Chile, Colombia, Costa Rica, Cuba, Dominica, Dominican Republic, Ecuador, El Salvador, Grenada, Guatemala, Guyana, Haiti, Honduras, Jamaica, Mexico, Nicaragua, Panama, Paraguay, Peru, Saint Kitts and Nevis, Saint Lucia, Saint Vincent and the Grenadines, Suriname, Trinidad and Tobago, United States, Uruguay, and Venezuela. WHO territories in AMR (nine): Anguilla, Aruba, Bermuda, British Virgin Islands, Cayman Islands, Curaçao, Montserrat, Sint Maarten, and Turks and Caicos Islands.

¶¶ WHO countries in EMR (21): Afghanistan, Bahrain, Djibouti, Egypt, Iran, Iraq, Jordan, Kuwait, Lebanon, Libya, Morocco, Oman, Pakistan, Qatar, Saudi Arabia, Somalia, Sudan, Syria, Tunisia, United Arab Emirates, and Yemen. WHO territories in EMR (one): occupied Palestinian territory, including east Jerusalem.

*** WHO countries in EUR (53): Albania, Andorra, Armenia, Austria, Azerbaijan, Belarus, Belgium, Bosnia and Herzegovina, Bulgaria, Croatia, Cyprus, Czechia, Denmark, Estonia, Finland, France, Georgia, Germany, Greece, Hungary, Iceland, Ireland, Israel, Italy, Kazakhstan, Kyrgyzstan, Latvia, Lithuania, Luxembourg, Malta, Monaco, Montenegro, Netherlands, North Macedonia, Norway, Poland, Portugal, Moldova, Romania, Russia, San Marino, Serbia, Slovakia, Slovenia, Spain, Sweden, Switzerland, Tajikistan, Turkey, Turkmenistan, Ukraine, United Kingdom, and Uzbekistan. WHO territories in EUR (one): Kosovo.

††† WHO countries in SEAR (11): Bangladesh, Bhutan, Burma, India, Indonesia, Maldives, Nepal, North Korea, Sri Lanka, Thailand, and Timor-Leste.

§§§ WHO countries in WPR (27): Australia, Brunei, Cambodia, China, Cook Islands, Federated States of Micronesia, Fiji, Japan, Kiribati, Laos, Malaysia, Marshall Islands, Mongolia, Nauru, New Zealand, Niue, Palau, Papua New Guinea, Philippines, South Korea, Samoa, Singapore, Solomon Islands, Tonga, Tuvalu, Vanuatu, and Vietnam. WHO territories in WPR (10): American Samoa, Hong Kong, Macau, French Polynesia, Guam, New Caledonia, Northern Mariana Islands, Pitcairn Islands Tokelau, and Wallis and Futuna.

¶¶¶ 45 C.F.R. part 46.102(l)(2), 21 C.F.R. part 56; 42 U.S.C. Sect. 241(d); 5 U.S.C. Sect. 552a; 44 U.S.C. Sect. 3501 et seq.

**** Belarus, Canada, Democratic Republic of the Congo, Guatemala, Haiti, Indonesia, Japan, Monaco, Morocco, Peru, Philippines, Russia, San Marino, Slovakia, Somalia, Timor-Leste, and the United States.

## References

[1] World Health Organization. Global vaccine action plan. Geneva, Switzerland: World Health Organization; 2013. https://www.who.int/teams/immunization-vaccines-and-biologicals/strategies/global-vaccine-action-plan

[2] World Health Organization. Meeting of the Strategic Advisory Group of Experts on Immunization, 31 March–1 April 2020: conclusions and recommendations. Wkly Epidemiol Rec 2020;22:241–56. https://apps.who.int/iris/bitstream/handle/10665/332218/WER9522-eng-fre.pdf?ua=1&ua=1

[3] World Health Organization. Immunization agenda 2030: a global strategy to leave no one behind. Geneva, Switzerland: World Health Organization; 2020. https://www.who.int/teams/immunization-vaccines-and-biologicals/strategies/ia2030

[4] World Health Organization. Report of the meeting of the WHO Global Advisory Committee on Vaccine Safety (GACVS), 1–3 December 2020. Wkly Epidemiol Rec 2021;96:13–20. https://www.who.int/publications/i/item/who-wer9603-13-20

[5] World Health Organization. Definition and application of terms for vaccine pharmacovigilance: report of CIOMS/WHO Working Group on Vaccine Pharmacovigilance. Geneva, Switzerland: World Health Organization; 2012. https://www.who.int/publications/m/item/9789290360834

[6] Salman O, Topf K, Chandler R, Conklin L. Progress in immunization safety monitoring—worldwide, 2010–2019. MMWR Morb Mortal Wkly Rep 2021;70:547–51. 10.15585/mmwr.mm7015a2 33857066 PMC8344995

[7] World Health Organization. Global vaccine safety blueprint. Geneva, Switzerland: World Health Organization; 2012. https://apps.who.int/iris/bitstream/handle/10665/70919/WHO_IVB_12.07_eng.pdf;sequence=1

[8] Uppsala Monitoring Centre. About VigiBase. Uppsala, Sweden: Uppsala Monitoring Center; 2023. https://www.who-umc.org/vigibase/

[9] World Health Organization. Immunization, vaccines, and biologicals: WHO/UNICEF joint reporting process. Geneva, Switzerland: World Health Organization; 2022. https://www.who.int/teams/immunization-vaccines-and-biologicals/immunization-analysis-and-insights/global-monitoring/who-unicef-joint-reporting-process

[10] World Health Organization. Safety. Geneva, Switzerland: World Health Organization; 2022. https://immunizationdata.who.int/pages/indicators-by-category/safety.html

